# Network dynamics in nanofilled polymers

**DOI:** 10.1038/ncomms11368

**Published:** 2016-04-25

**Authors:** Guilhem P. Baeza, Claudia Dessi, Salvatore Costanzo, Dan Zhao, Shushan Gong, Angel Alegria, Ralph H. Colby, Michael Rubinstein, Dimitris Vlassopoulos, Sanat K. Kumar

**Affiliations:** 1 Foundation for Research and Technology - Hellas (FORTH), Institute of Electronic Structure and Laser, Heraklion, Crete 70013, Greece; 2 Department of Materials Science and Technology, University of Crete, Heraklion, Crete 71003, Greece; 3 Department of Chemical Engineering, Columbia University, New York, New York 10027, USA; 4 Department of Materials Science and Engineering, Pennsylvania State University, University Park, Pennsylvania 16802, USA; 5 Departamento de Física de Materiales y Centro de Física de Materiales CSIC-UPV/EHU, Universidad del País Vasco UPV/EHU, San Sebastian E-20018, Spain; 6 Department of Chemistry, University of North Carolina, Chapel Hill, North Carolina 27599-3290, USA

## Abstract

It is well accepted that adding nanoparticles (NPs) to polymer melts can result in significant property improvements. Here we focus on the causes of mechanical reinforcement and present rheological measurements on favourably interacting mixtures of spherical silica NPs and poly(2-vinylpyridine), complemented by several dynamic and structural probes. While the system dynamics are polymer-like with increased friction for low silica loadings, they turn network-like when the mean face-to-face separation between NPs becomes smaller than the entanglement tube diameter. Gel-like dynamics with a Williams–Landel–Ferry temperature dependence then result. This dependence turns particle dominated, that is, Arrhenius-like, when the silica loading increases to ∼31 vol%, namely, when the average nearest distance between NP faces becomes comparable to the polymer's Kuhn length. Our results demonstrate that the flow properties of nanocomposites are complex and can be tuned via changes in filler loading, that is, the character of polymer bridges which ‘tie' NPs together into a network.

## Abstract (web summary)

The mixture of nanoparticles and polymers can dramatically alter the dynamics of the resulting system, but the detail is still under debate. Here, Baeza *et al*. show the formation of a percolated network by polymer bridging between adjacent nanoparticles as nanoparticle concentration increases.

## Graphical abstract

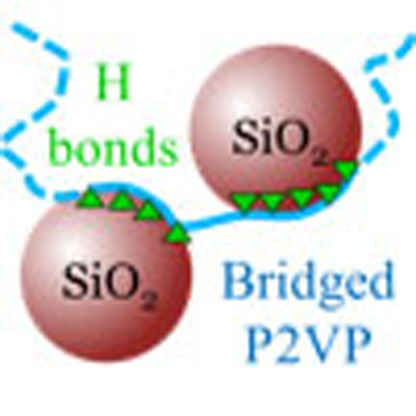

The ability of nanoparticles (NPs) to substantially alter the macroscopic properties of polymers to which they are added is well established, but developing the ability to improve properties, for example, mechanical, remains an open challenge^1^,^2^,^3^,^4^,^5^,^6^,^7^,^8^,^9^,^10^,^11^,^12^,^13^,^14^,^15^,^16^,^17^. Restricting the discussion to spherical NPs, it is accepted that particle size and loading influence the local (segmental *α*-relaxation) dynamics of the matrix polymer^12^,^13^,^18^,^19^, and there is consensus that the region in the neighbourhood of the NP, sometimes termed the ‘bound' layer, has reduced segmental mobility, driven by attractive NP-polymer interactions. Long and co-workers go further and suggest that favourable NP-polymer interactions lead to the formation of a glassy (that is, dynamically frozen) bound polymer layer^14^,^20^. While the existence of a bound polymer layer has now been verified^12^,^21^,^22^,^23^, it is also established that its segmental dynamics is not necessarily glassy^24^,^25^. There is therefore good understanding of segmental dynamics in these situations.

In contrast, an outstanding challenge is the consequence of these segmental dynamics on collective system relaxation, and hence mechanical reinforcement, especially at high NP loadings. There are two dominant trains of thought in the field: first, mechanical reinforcement is purely due to NP clusters^26^ and second, there is a network formed with the NPs as the nodes and the adsorbed polymers as the network strands^27^. Long and co-workers further suggest that mechanical reinforement results when the glassy layers on adjacent NPs overlap, and the resulting network structure percolates^14^,^20^,^28^,^29^. Apart from the new feature of a glassy bound polymer layer, this last picture is analogous to the pure NP aggregation mechanism discussed above. Since it is not clear if bound glassy layers are always formed at attractive NP/polymer interfaces, their importance in mechanical reinforcement and how they can affect system dynamics of nanofilled polymers remains open.

We address this challenge here. In particular, we demonstrate that system dynamics at low NP loadings are polymer-like, but with an increased friction, presumably due to favourable NP-polymer interactions. Above a critical NP loading, specifically when the mean inter-NP spacing becomes smaller than the polymer's entanglement tube diameter, they become consistent with the formation of a polymer-mediated NP network. System dynamics are gel-like in this regime, but the temperature dependence of system relaxation still follows the Williams–Landel–Ferry (WLF) relation reminiscent of the polymer chains. This behaviour turns into a particle-dominated Arrhenius-like dependence at even higher loadings, that is, where the NP face-to-face distances are comparable to the Kuhn length of the chains (≈1 nm, ref. 30). Thus, when the NPs with their tightly bound polymer layers begin to overlap, system dynamics lose any obvious connections to those of the polymer chains that bridge the NPs. These novel findings unequivocally establish network formation as the cause of mechanical reinforcement in this class of polymer nanocomposites, and suggest that it could be due to either of the two mechanisms proposed in the literature in different regions of parameter space.

## Results

### Systems studied

We investigate canonical, favourably interacting mixtures of silica NPs (Nissan MEK-ST, of a diameter *d*=14±4 nm) with poly(2-vinylpyridine) (P2VP, *T*_g_≈375 K), of molecular weights *M*_w_=554, 219, 105 and 36 kg mol^−1^, and respective radii of gyration *R*_g_ of 17.8, 11.2, 7.7 and 4.5 nm, using a suite of static and dynamic probes. These nanocomposites have an essentially uniform distribution of NPs in the polymer matrix at all loadings, a result that has been established using transmission electron microscopy (TEM; Supplementary Fig. 1)^12^,^13^,^31^,^32^,^33^. Using TEM alone to characterize NP dispersion is unsatisfactory due to the small sample sizes (typically several μm on a side) coupled to the thickness of the microtomed slices (typically 60 nm in our work). Thus, NPs which are at different ‘depths' in a sample can appear to be overlapping in a TEM image. These issues are alleviated by the use of a scattering probe, such as ultrasmall angle X-ray scattering, which we previously used to show that the NPs do not cluster^32^; in addition, the mean distance between the NPs deduced from these measurements are consistent with uniform NP spatial dispersion. Consequently, these NPs are well-dispersed at all scales that we can characterize^32^. Moreover, while two-dimensional NMR (2D-NMR) shows that no chemical reactions occur during sample preparation or during the subsequent thermal annealing, Fourier transform infrared spectroscopy (FTIR) measurements indicate strongly favourable interactions between the NPs and the polymer^22^, that is, strong hydrogen-bonds (H-bonds) between the silanol groups on the particle surface and the nitrogen atoms of the pyridine ring in the polymer backbone (see the FTIR and NMR sections, Supplementary Figs 2–5).

### Segmental dynamics

**Figure 1:**
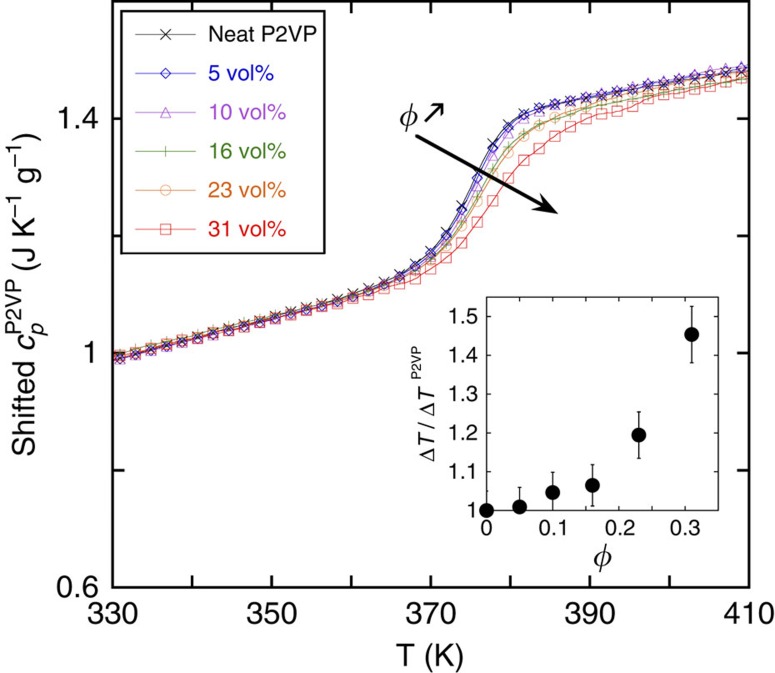
Glass transition broadening in nanocomposites. Specific heat capacity of the polymer fraction as a function of the temperature for neat P2VP (554 kg mol^−1^) and nanocomposites loaded with 5, 10, 16, 23 and 31 vol% silica. 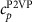 is calculated by subtracting the silica contribution from the nanocomposite's heat capacity as calculated in ref. 13. Note that the thermograms are arbitrarily shifted to illustrate the broadening of the glass transition. The inset is the breadth Δ*T* of the glass transition as a function of the filler fraction normalized by that of the neat P2VP. The error bars come from the temperature range considered around the glass transition to establish linear dependency of the heat capacity.

Figure 1 shows differential scanning calorimetry (DSC) results of nanocomposite samples with the 554 kg mol^−1^ polymer matrix as a function of NP loading. Note that in the following text the volume fraction *φ* is the ratio of the volume of silica NPs to the total sample volume. Here we plot the specific heat capacity of the polymer^13^ as a function of temperature. While the calorimetric glass transition temperature *T*_g_ of the P2VP matrix (375 K) is barely affected by NP loading, the transition becomes broader, especially for NP loadings of 16 vol% and larger. In fact, the breadth of this transition goes from 11 K for pure P2VP to 11.5 K for 16 vol% and 16 K for 31 vol% NP loading, that is, 1.5 times broader than the neat matrix, regardless of *M*_w_ (see inset of Fig. 1 and Supplementary Fig. 6). Apparently, segmental dynamics become more heterogeneous with increased NP loading, with significant changes occurring somewhere between 16–31 vol% NPs^30^.

**Figure 2:**
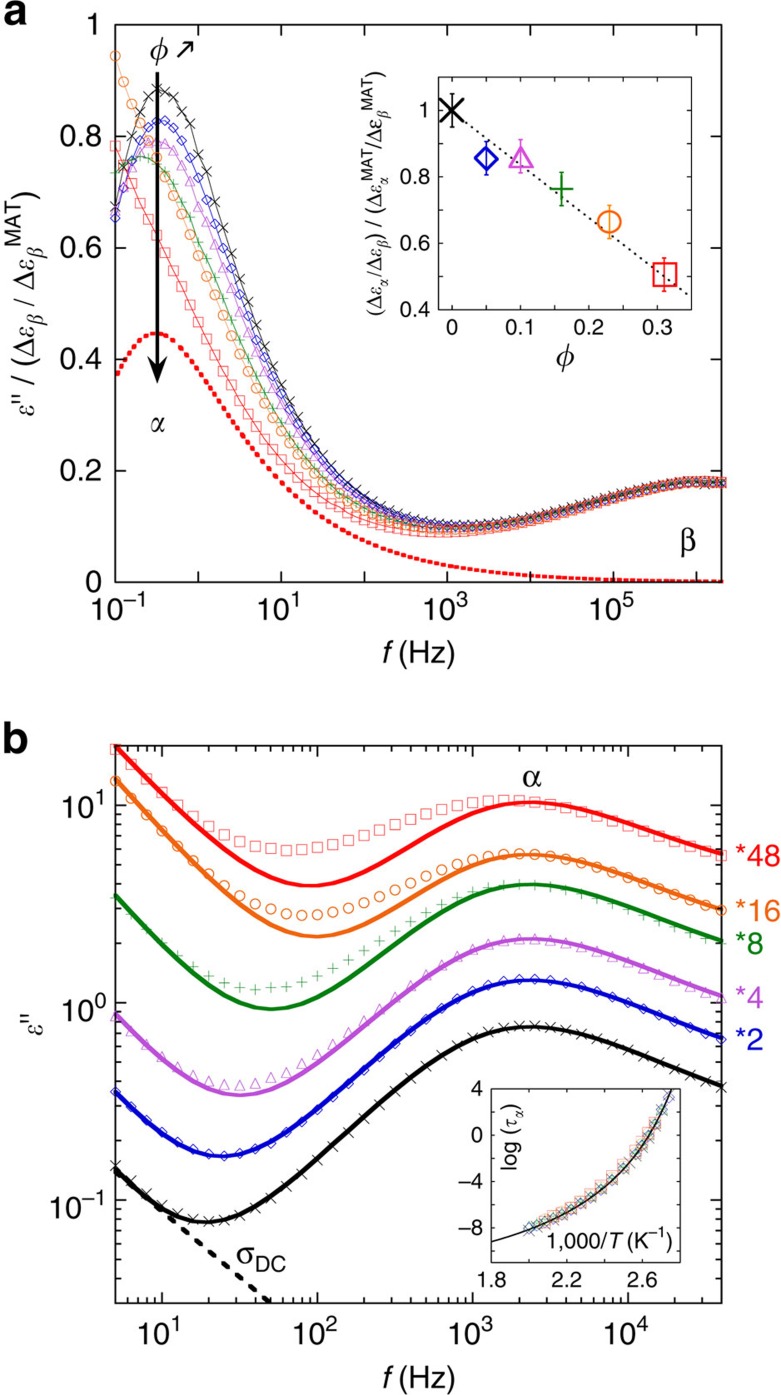
Dielectric properties of nanocomposites. (**a**) Dielectric loss (*ɛ*″) reduced by the ratio of the *β*-process intensity over the corresponding matrix one, 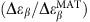, as a function of frequency at *T*=380 K for neat P2VP (cross) and nanocomposites loaded with 5 (diamond), 10 (triangle), 16 (plus), 23 (circle) and 31 (square) vol%. The red dashed line is the contribution of the *α*- process to the global *ɛ*″ in the 31 vol% sample. The inset is the ratio of the *α-* and *β*-relaxation strength normalized by the corresponding quantity for the pure matrix 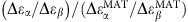 as a function of the NP loading. The decreasing trend highlights the relative attenuation of the *α*-relaxation with the NP loading. (**b**) Similar measurements performed at *T*=410 K have been vertically shifted for clarity (see numbers on the right side). Solid lines are fits to the data with a *σ*_DC_ contribution at low frequency and a Havriliak–Negami function with a fixed *α*-relaxation shape (symmetric and asymmetric), as found in the neat matrix in order to illustrate its broadening when NP content is increased. *σ*_DC_ (black dashed line) stands for the contribution of the ionic conductivity. Inset: segmental relaxation time log(*τ*_*α*_) as a function of 1,000/*T*. The solid line is fit to the neat P2VP data with the WLF equation.

The local P2VP dynamics are also characterized using dielectric spectroscopy^12^,^13^. Figure 2 shows that NP loading has a strong influence on the strength of the segmental *α*-relaxation process, which decreases at high silica content; the NPs also broaden this relaxation towards lower frequencies (Fig. 2b), in good agreement with the DSC data. However, the mean relaxation time of the *α*-process, *τ*_*α*_, remains essentially unaltered (see inset of Fig. 2b) as does the mean time of the more local, *β*-relaxation (see Supplementary Figs 7–9)^18^. The broadening of the *α*-process with increased NP loading is consistent with the DSC results and is attributed to the bound polymer layer formed due to strong NP-polymer interactions^12^,^14^,^18^.

### Collective dynamics

**Figure 3:**
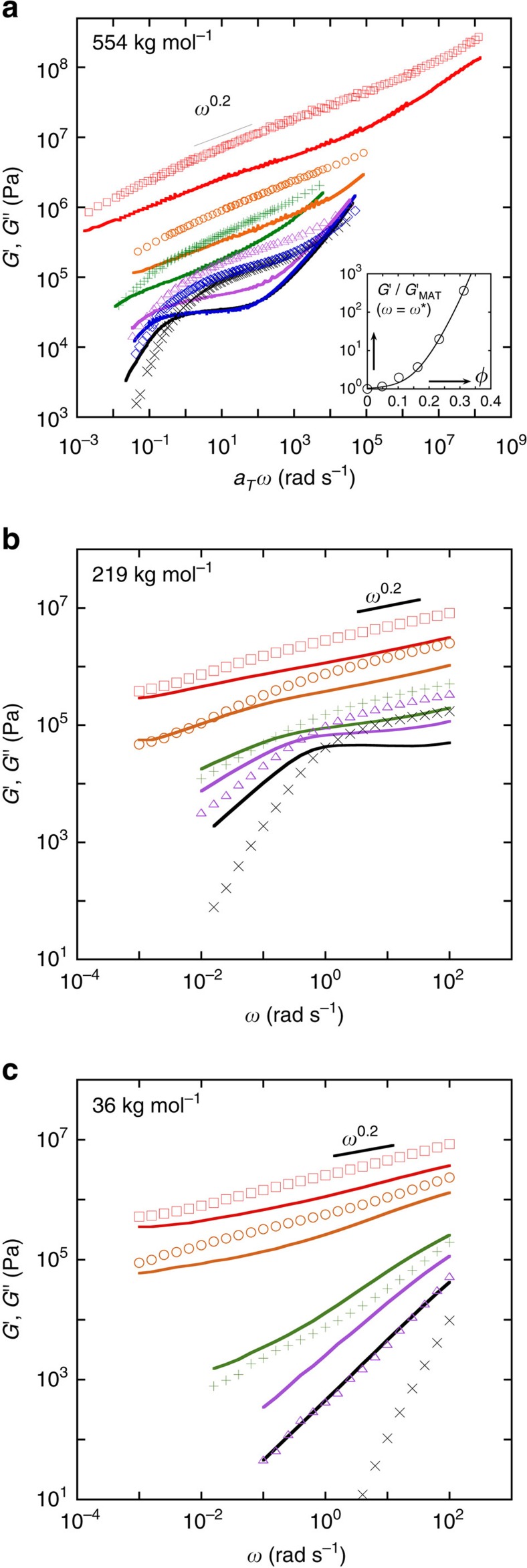
Rheological behaviour of the nanocomposites. (**a**) Master curves of storage (*G*′, symbols) and loss (*G*″, solid lines) moduli as a function of frequency at *T*_ref_=473 K for neat P2VP 554 kg mol^−1^ (cross) and nanocomposites loaded with 5 (diamond), 10 (triangle), 16 (plus), 23 (circle) and 31 (square) vol% in silica. The master curves were obtained by frequency-scale shifts only. Inset: relative increase of the storage modulus as a function of the silica volume fraction for *ω* where tan*(δ*)=*G*″/*G*′ reaches its minimum (Supplementary Fig. 10). The solid line is a fit to the Doolittle equation^41^ with *A*=0.052 and *B*=19.46 (see text). (**b**,**c**) Respective measurements performed in different matrices at a single temperature *T*=453 K for (**b**) *M*_w_=219 kg mol^−1^ and (**c**) *M*_w_=36 kg mol^−1^. The legends are the same as that in **a**, where the symbols and solid lines respectively stand for *G*′ and *G*″.

Viscoelastic master curves in the linear regime (storage *G*′ and loss *G*″ moduli plotted against reduced angular frequency *a*_*T*_*ω*) obtained using time–temperature superposition (Supplementary Figs 10 and 11) at each NP loading are compiled in Fig. 3a for the 554 kg mol^−1^ matrix. (Supplementary Fig. 12 verifies that these data are collected in the regime of linear response.) Limited, single temperature (*T*=453 K) data for nanocomposites based on shorter P2VP chains (219 and 36 kg mol^−1^) are also shown for comparison (Fig. 3b,c, respectively). Up to 10 vol% NP (Fig. 3a), a response typical of viscoelastic liquids is found with the following additional observations: the entanglement plateau modulus (of pure P2VP) is barely affected by the addition of 5 vol% silica, whereas the clear increase at 10 vol% reflects the reinforcing action of NPs (for example, the *G*′ at a frequency of 100 rad s^−1^). Moreover, the local dynamics (the high-frequency moduli crossover, which is associated with the Rouse time of an entanglement strand for pure entangled polymers) is unaffected. The NPs thus simply serve to increase the friction, as might be expected by the venerable Einstein-Sutherland-Batchelor ideas.

With increasing NP loading, the ratio of high- to low-frequency moduli crossover increases (that is, the width of the intermediate plateau-like region extends to a wider range of frequencies, Fig. 3a). We find ratios of 1.83 × 10^4^ for pure P2VP, 5.08 × 10^4^ for 5 vol%, 1.56 × 10^5^ for 10 vol% and 3.52 × 10^6^ for 16 vol% NP. Above 16 vol%, it appears that the nanocomposites behave like gels over a wide frequency range down to the lowest frequencies probed (Fig. 3). Since these observations are independent of the molecular weight of the matrix chains (to first order), we cannot evoke the chain size (and its relationship to the inter-NP spacing) as being the cause of this effect. We remark that, at 16 vol%, the mean NP-NP face-to-face separation (about 12 nm, calculated following 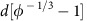) becomes comparable to the tube diameter of this polymer (which is estimated to be 13–15 nm from the molecular weight between entanglements, *M*_e_, extracted from Supplementary Fig. 13). At 23 vol% the network becomes denser (we estimate the distance from the face of a NP to the face of its nearest particle is ∼2.6 nm, while the mean distance between NP faces is ∼9 nm, see Monte-Carlo simulations section in Supplementary Information) and the high-frequency crossover in the master curve (Fig. 3a) disappears, suggesting that the system rheology cannot be depicted through chain dynamics in the framework of the tube model. At these high NP loadings, it appears that adsorption interactions between the NPs and the polymers hinder chain motion, rather than the standard chain–chain entanglements which occurs in the molten state^19^,^34^.

More importantly, with increasing NP loading (from 16% to 31 vol%), the storage modulus in the intermediate frequency region exhibits a power law response with an approximate scaling *G*′∼*ω*^0.2^, practically independent of the P2VP molar mass. The 31 vol% sample exhibits the same power law dependence over a wide frequency range (about 10 decades down to 10^−2^ rad s^−1^). At lower frequencies (10^−3^ to 5 × 10^−5^ rad s^−1^, see data obtained from creep measurements, Supplementary Fig. 14), however, there is a transition in behaviour with a self-similar response approximately characterized by a power law exponent of 0.5 (ref. 35). Finally, at the lowest frequency, *G*′ tends towards a plateau while *G*″ keeps decreasing. A few facts that arise from this data are noted: the *G*′∼*ω*^0.2^ behaviour which is observed over a large frequency range reflects the slow motion of short P2VP chain segments, which are adsorbed at the surface of the particles as ‘trains' and require an exponentially distributed spectrum of desorption modes. Eventually there will be a change of slope due to predominance of desorption, but no flow (see Supplementary Information for further details). Additionally, the observed reinforcement (inset of Fig. 3a) is well fitted by the empirical Doolittle equation, that is, 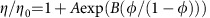 (where *η*_0_ and *η* are the viscosity of the pure polymer and the nanocomposite, respectively, *φ* is the NP volume fraction, and *A*, *B* are fit parameters), which is often used to describe the viscosity *η* of glass forming materials based on free volume considerations. Finally, a time sweep measurement (Supplementary Fig. 15) reveals that the nanocomposite loaded with 31 vol% silica shows extremely delayed relaxation (beyond the experimental measurement window) consistent with the extended frequency spectrum presented in Supplementary Fig. 14b. This suggests that the structure obtained is temporally evolving as in transient gels or glasses (this is the well known physical aging mechanism in kinetically arrested systems). These independent observations support the same idea: the nanocomposites show a dynamic transition when the NP loading is increased to 31 vol%. We shall speculate below on the structural origins of this transition.

**Figure 4:**
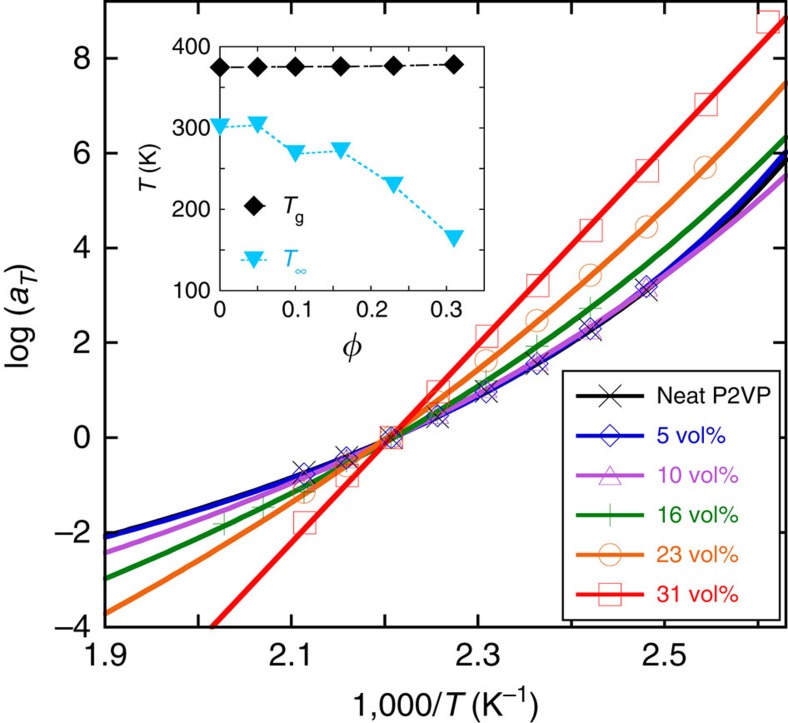
Variations of shift factors with temperature and NP loading. Temperature dependence of the frequency-scale shift factor *a*_*T*_ used to build the viscoelastic master curves in Fig. 3a for the neat P2VP 554 kg mol^−1^ matrix and its associated nanocomposites. Solid lines are WLF fits to the data, except for the 31 vol% data, which is fit to an Arrhenius equation; fit parameters are provided in Supplementary Table 1. Inset: NPs volume fraction dependence of the glass transition temperature *T*_g_ and Vogel temperature *T*_∞_ for the same samples. *T*_∞_ has been extracted from fitting the data of the main figure with the Vogel–Fulcher–Tammann equation (see Supplementary Table 1).

The essential information on the temperature dependence of the measured chain-level dynamics is summarized in Fig. 4, which depicts the frequency-scale shift factors as a function of inverse temperature for the different NP loadings studied, using *T*_ref_=453 K (see also Supplementary Table 1 and Supplementary Fig. 16). Note that the temperature ranges we can explore are limited from below by the vitrification of the polymer (∼375 K, independent of loading), and from above by thermal degradation, which occurs beyond 473 K. First, we note that the shift factors obtained from the different NP loadings do not superimpose. More interestingly, at 31 vol% a clear change from WLF to Arrhenius behaviour is observed, with the latter characterized by an unusually large activation energy (403 kJ mol^−1^). Concominantly, the extrapolated Vogel temperature, *T*_∞_, decreases strongly with NP loading and its distance from *T*_g_ is much larger at 31 vol% (inset of Fig. 4). In fact, the growing difference between *T*_g_ and *T*_∞_ with increasing *φ* implies that polymer–polymer interactions become less important relative to global dynamics. While the Arrhenius temperature dependence may thus be rationalized by the experiments being performed at temperatures far above the Vogel temperature, this result appears at odds with the fact that these materials show very slow relaxation and age (gel/glassy behaviour). These facts can be reconciled by postulating that the material behaves akin to a percolating NP-polymer network, but with a slow local rearrangement of adsorbed chains (Supplementary Fig. 15); the Arrhenius dependence reflects the (apparently non-cooperative) activated exchange of adsorbed segments with bulk molecules. These experiments have been repeated on shorter P2VP chains (*M*_w_=105 kg mol^−1^; see Supplementary Figs 17 and 18) where similar results have been found.

The outstanding question then is the structural basis of the transition seen in the temperature dependence of system dynamics at a loading of 31 vol%. Computer simulations of hard sphere fluids have been used to calculate the mean face-to-face distances to the first, second and third nearest neighbouring NPs as a function of loading (Supplementary Tables 2 and 3). Between 23 and 31 vol% it is apparent that the distance to the closest neighbour drops to ∼1.65 nm (i.e., about 1 to 2 Kuhn lengths). Under these circumstances, many of the nearest NP faces must accommodate only one Kuhn segment; in other words, the NPs come so close that they are bridged by one bound Kuhn segment, which by definition is adsorbed simultaneously on both NPs. It is the accompanying doubling of the (free) energy of desorption that we believe exponentially increases the lifetime of these bridges. Similarly, the second nearest NP is only 3 Kuhn segments away. This corresponds to one Kuhn segment adsorbed on each NP, with a third, strongly constrained internal segment. Since each of these segments adsorb/desorb effectively independent of each other, there should be no cooperative effects associated with their slow relaxations. It is these two facts, in combination, that we believe are responsible for the Arrhenius dependence that emerges under these larger NP loadings^36^.

We now rationalize the large activation energy. It is true that, even though most of polymers exhibit an Arrhenius-like dependence of viscosity at temperatures far above *T*_g_ (typically at *T*_g_+150 K or *T*_∞_+200 K), the corresponding activation energy is usually much lower than that found in our case. For example, metallocene-based polyethylenes have an activation energy of ∼35 kJ mol^−1^ (ref. 37). We conjecture that the large activation energy seen is a consequence of favourable silica-P2VP interactions (for the silica-P2VP bond see sections of FTIR and 2D-NMR in Supplementary Information). At high NP loadings (>10 vol%), this results in the percolation of NPs and hence a NP-polymer network forms^30^. For the 31 vol% loading, where face-to-face contacts are mediated by a Kuhn segment, we conjecture that the relaxation dynamics are dominated by the release of this doubly adsorbed Kuhn segment. With an average of 4–5 such ‘doubly adsorbed' P2VP Kuhn segments per NP (a reasonable hypothesis), and a N–H bond activation energy of ∼45 kJ mol^−1^ (ref. 38) (or 90 kJ mol^−1^ for detachment of a Kuhn segment), this results in an energy of ≈400 kJ mol^−1^, that is, close to what is experimentally found. The FTIR data provided in Supplementary Figs 3 and 4 supports this strong silica-P2VP bonding through the perturbation of the Si–OH bonds present at the NPs surface. Further evidence comes from viscoelastic measurements at higher strains (Supplementary Fig. 19) which indicate that the activation energy is reduced as strain increases, apparently due to some strain-induced debonding of the NP-polymer bound layer, in agreement with literature reports^39^. Finally, simple dissolution experiments corroborate this scenario. We find that while P2VP dissolves in chloroform, the high NP loading nanocomposites do not. On the other hand, both matrix and nanocomposite dissolve in THF which is a hydrogen bond acceptor, apparently due to substitution of silanol-P2VP by THF-P2VP hydrogen bonding.

In summary, in our system the average local segmental level dynamics (*α*-process, which presumably reflects the motions of the unadsorbed chain segments) show no change with the added NPs although it becomes broader at higher loadings, supporting the fundamental decorrelation of dynamics at different length scales as already observed by Ding and Sokolov^40^. Mechanical spectroscopy shows a clear transition in system dynamics, from that corresponding to the slower relaxation of chains (due to the presence of adsorbing NPs) at low NP loadings, to that of a network when the NP loading exceeds a critical value of ∼10–16 vol% (with essentially no molecular weight dependence). Following past work^27^, we deduce that a particle-dominated network comprised of silica NPs ‘connected' through adsorbed P2VP chains forms at these higher NP loadings. Through geometrical arguments we deduce that this transition occurs when the mean face-to-face distance between the NPs becomes comparable to the entanglement tube diameter of the neat polymer (*a*≈13–15 nm). More interestingly, the temperature dependence of system dynamics changes to Arrhenius-like for the 31 vol% sample, that is, when the closest face-to-face distance between two NPs becomes comparable to the Kuhn length of the polymer chains in question (*b*≈1 nm). In these cases, the shortest mean bridge comprises only one adsorbed Kuhn segment, while the second shortest bridge is formed by three Kuhn segments; we propose that their sluggish desorption dynamics is reflected in the Arrhenius temperature dependence of the experimental relaxation data. These results demonstrate both the onset of network-like behaviour (gel-like in a wide range of frequency but not permanently percolated) in these systems, but, more pertinently, they show that its character can be readily tuned through variations of the NP loading. Our novel findings offer a unified explanation that network formation, that is NPs bridged by bound polymer segments, dominates the mechanical behavior of this class of polymer nanocomposites.

## Methods

### Nanocomposites preparation and processing

All materials were used as received. Methyl ethyl ketone (MEK, HPLC-grade, >99.7%) and pyridine (ACS agent, >99.0%) were purchased from Sigma Aldrich (USA). P2VP (*M*_*w*_=554 kg mol^−1^, *M*_*w*_/*M*_*n*_=1.12; *M*_*w*_=105 kg mol^−1^, *M*_*w*_/*M*_*n*_=1.08) was obtained from Polymer Source. P2VP (*M*_*w*_=36 kg mol^−1^, *M*_*w*_/*M*_*n*_=1.07 and *M*_*w*_=219 kg mol^−1^, *M*_*w*_/*M*_*n*_=1.11) was ordered from Scientific Polymer Products. The colloidal silica NPs (MEK-ST) and the antioxidant Irganox 1010 were donated by Nissan Chemical Industries and Ciba Specialty Chemicals, now BASF, respectively.

Nanocomposite films were prepared by co-casting composite dispersions of colloidal silica NPs and P2VP in a mixture of MEK and pyridine. First, the as-received Nissan silica suspensions were diluted with pyridine in a volume ratio of 4:1. In the meantime, as-received P2VP and antioxidant Irganox (0.2 wt% relative to P2VP) solutions in MEK were prepared. Following that, appropriate amounts of diluted silica dispersion were added to the polymer/Irganox solution, resulting in silica/P2VP formulations with a known weight ratio (see TGA; Supplementary Fig. 20). For high molar-mass polymer-based samples, especially at high silica loadings, additional small amount of pyridine was gradually added into the composite suspensions to dissolve the bridged silica/P2VP precipitants back into the solution. The resulting mixtures were vortex-shaken for 2 h and then probe ultrasonicated for 3 min using an Ultrasonic Processor (model GEX-750) operated at 24% of maximum amplitude with a pulse mode of 2 s sonication following by 1 s rest time. The solutions were then poured into a PTFE Petri dish and air dried in a fume hood for at least 5 days. Finally, in order to provide the identical sample history and completely remove all the residual solvents in the films, the as-cast samples were annealed following a well-defined procedure: 7 days at 353 K and then 10 days at 423 K, both under vacuum. The films were milled down and remoulded at 453 K and 1 bar in a hot press equipped with a vacuum mould, for 15 h (463 K and 2 bars for the 31 vol% sample). This additional step allowed specimens of 8 mm in diameter and 0.5–0.6 mm in thickness to be produced. DSC and dielectric spectroscopy tests have been subsequently performed to verify that this additional processing step did not change local segmental properties. The experimental methods are described in the Supplementary information.

## Additional information

**How to cite this article:** Baeza, G. P. *et al*. Network dynamics in nanofilled polymers. *Nat. Commun.* 7:11368 doi: 10.1038/ncomms11368 (2016).

## Supplementary Material

Supplementary Information Supplementary Figures 1-20, Supplementary Tables 1-3, Supplementary Notes 1-8, Supplementary Methods and Supplementary References.
