# Supplementary material for: Network dynamics in nanofilled polymers
Source: Nat Commun. 2016 Apr 25;7:11368. doi: 10.1038/ncomms11368 (PMC4848491; doi:10.1038/ncomms11368)
Supplement: Supplementary Information — Supplementary Figures 1-20, Supplementary Tables 1-3, Supplementary Notes 1-8, Supplementary Methods and Supplementary References. [file ncomms11368-s1.pdf]

## Supplementary Figures

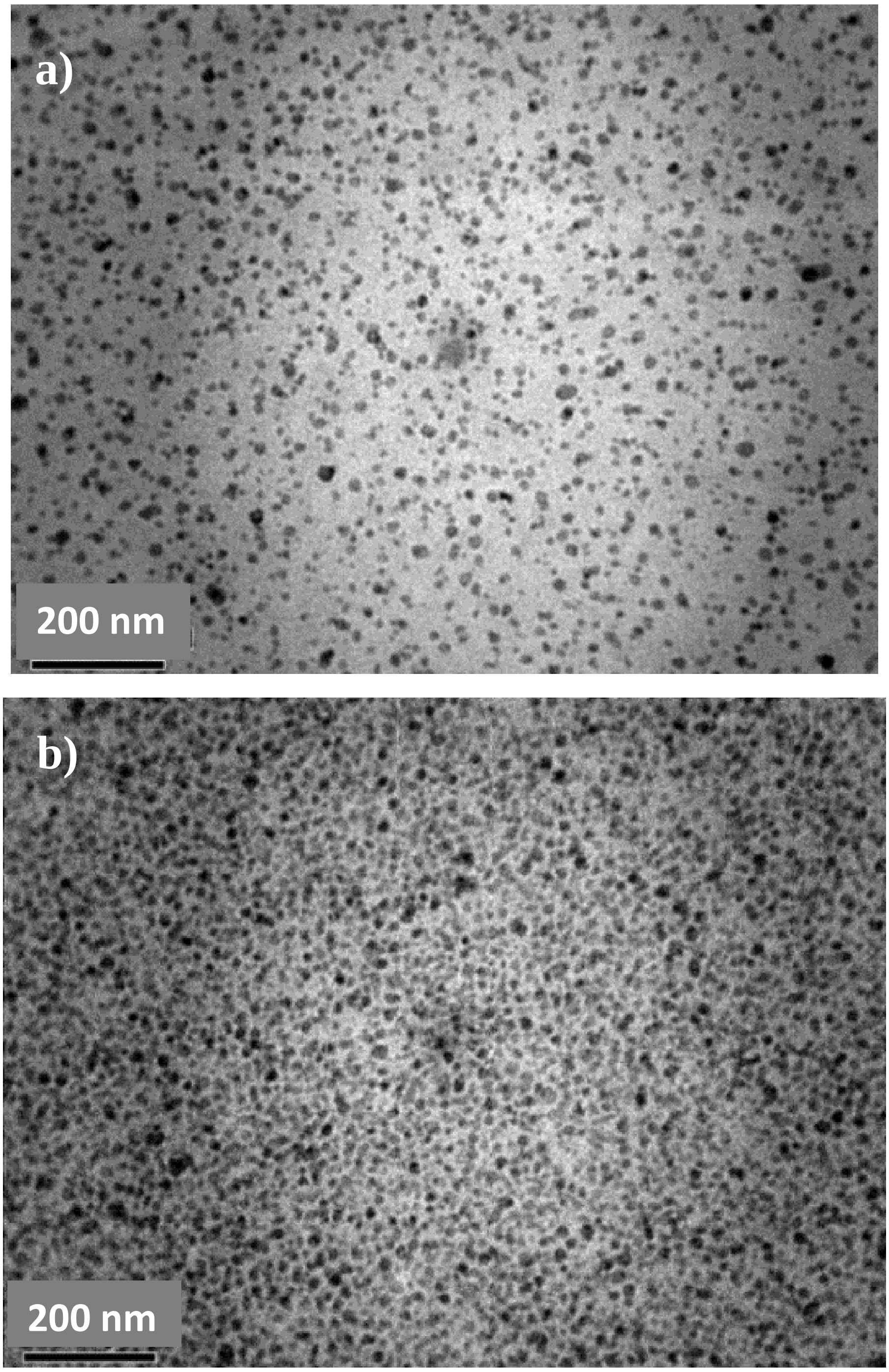


**Supplementary Figure 1**. TEM micrographs of nanocomposites cast in a mixture of MEK and pyridine loaded with **a)** 5 vol% and **b)** 31 vol% silica NPs.

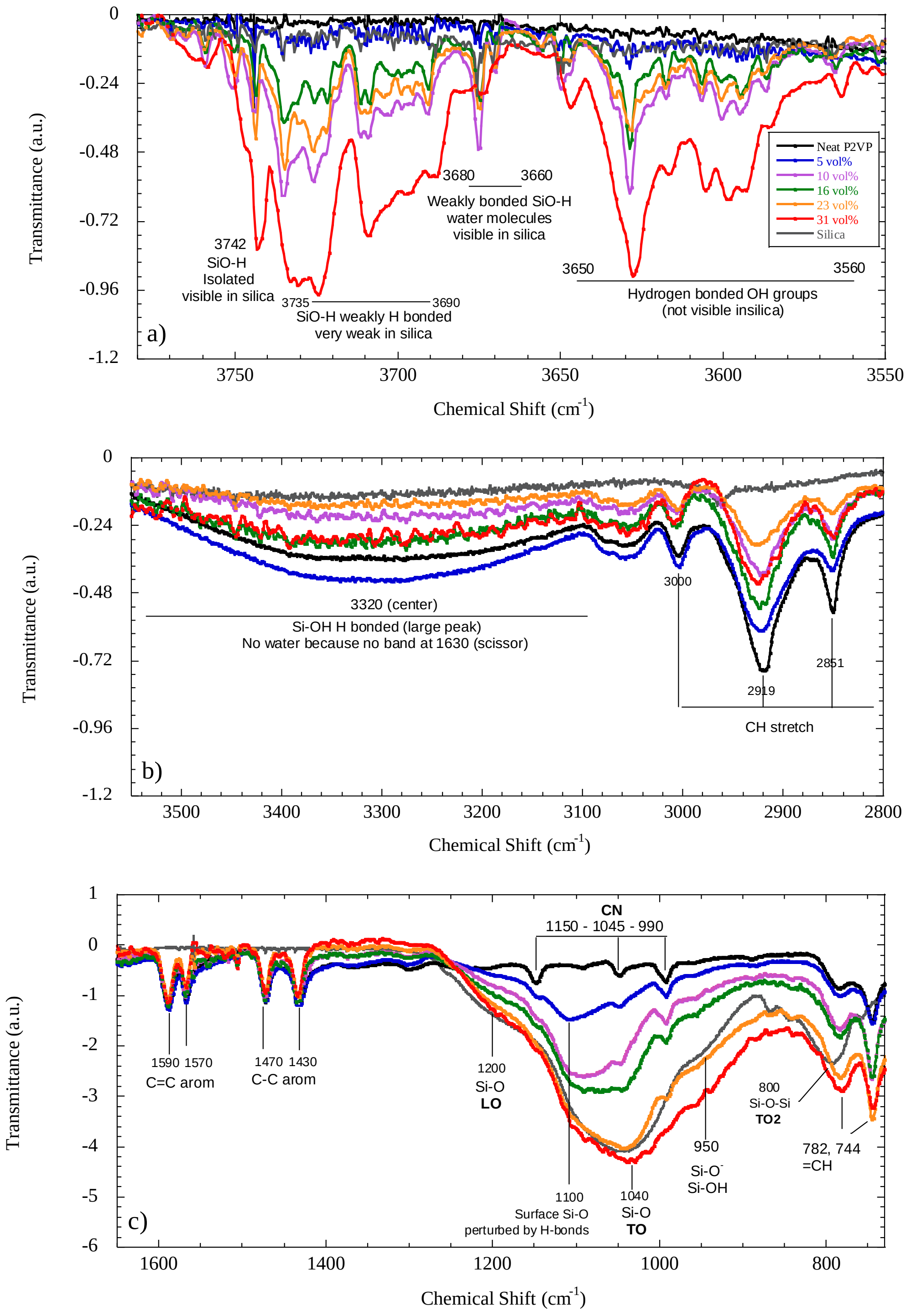


**Supplementary Figure 2.** Fourier Transform Infra-Red spectroscopy spectra of the neat P2VP ($M_w$ = 554 kg mol$^{-1}$) and the corresponding nanocomposites as well as the bare silica (grey). **a)** 3770-3550 cm$^{-1}$, **b)**3550-2800 cm$^{-1}$ and **c)** 1650-750 cm$^{-1}$.

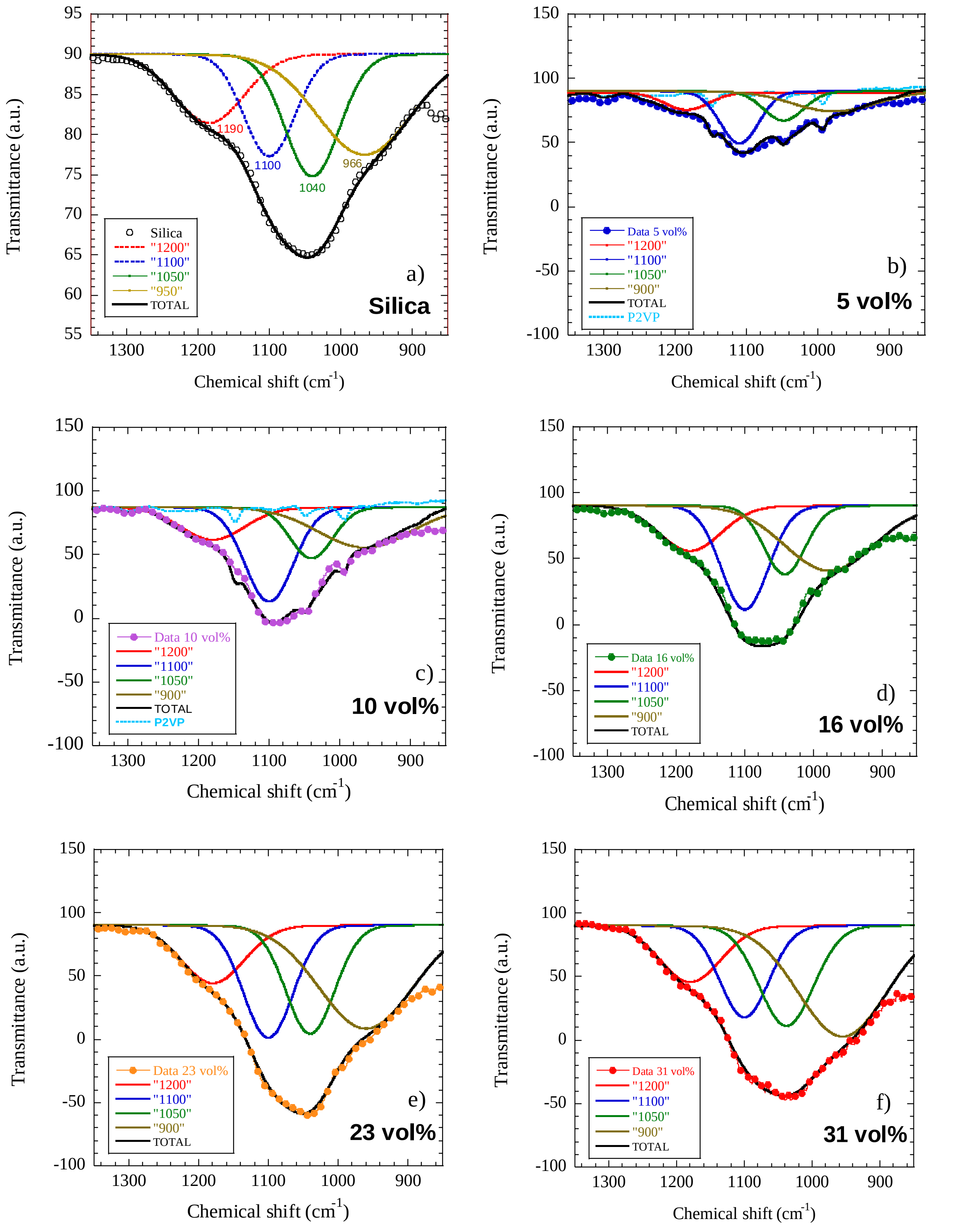


**Supplementary Figure 3.** Tentative of deconvolution of the Si-O « multi-peak » with four Gaussian contributions. **a)** bare silica, **b)** 5 vol%, **c)** 10 vol%, **d)** 16 vol%, **e)** 23 vol% and **f)** 31 vol%.

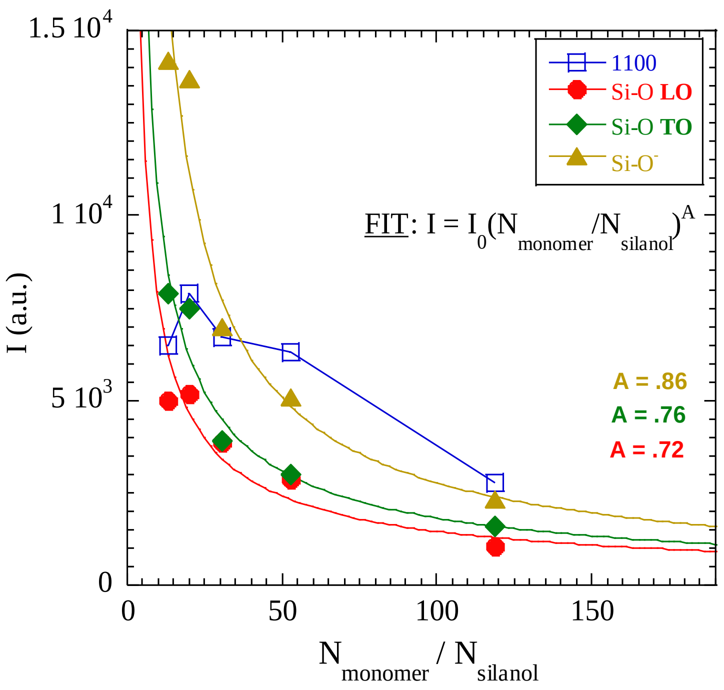


**Supplementary Figure 4.** Intensities of the different contributions used to fit the Si-O multi peak in FTIR spectra as a function of the silica content expressed as $N_{monomer}/N_{silanol}$ (considering 4 silanols per $nm^2$).

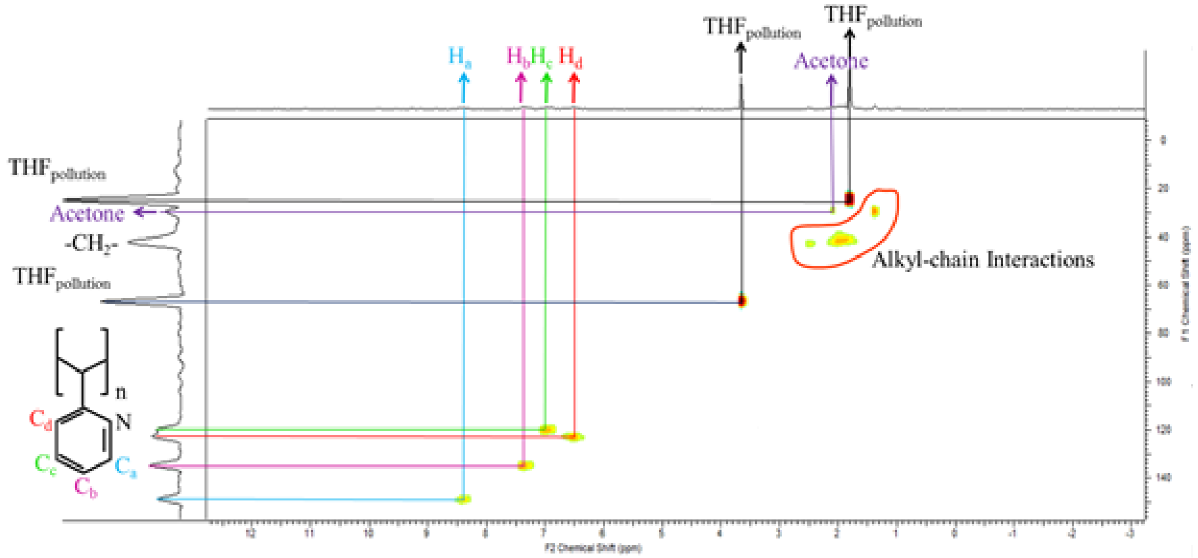


**Supplementary Figure 5.** 2D NMR ($^2H$, $^{13}C$) performed on the nanocomposite loaded with 31 vol% in silica with $M_w$ = 554 kg mol$^{-1}$.

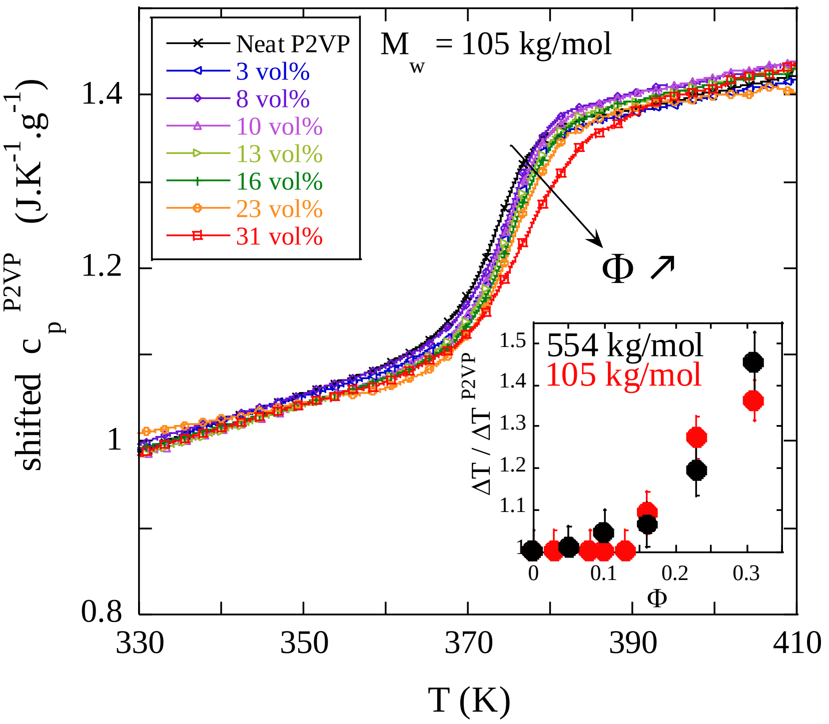


**Supplementary Figure 6.** Specific heat capacity as a function of the temperature for a neat P2VP ($M_w$ = 105 kg mol-1) and the corresponding nanocomposites loaded with 3, 8, 10, 13, 16, 23 and 31 vol%. The insert represents the breadth of the glass transition $\Delta T$ as a function of the filler fraction normalized by that of the neat P2VP for $M_w$ = 554 kg mol-1 (black – same as Fig. 1 in the article) and 105 kg mol-1 (red). See Fig. 1 in the article for error bars.

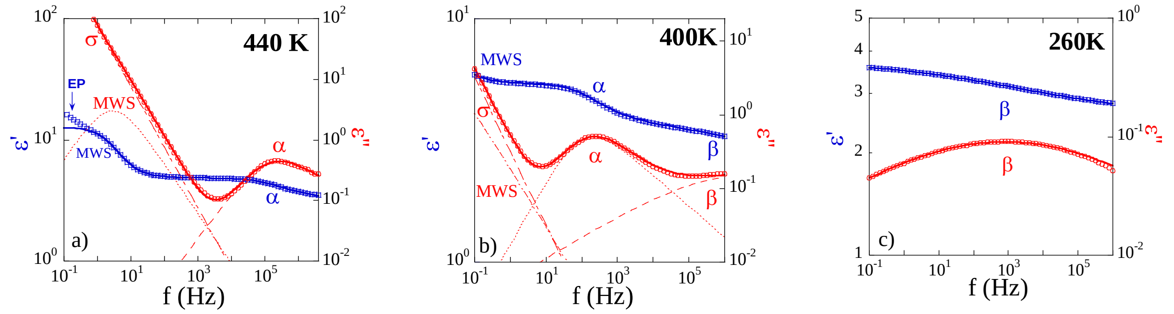


**Supplementary Figure 7.** Dielectric spectra of the composite loaded with 16 vol% cast in a mixture of MEK and pyridine measured at **a)** 440 K, **b)** 400 K and **c)** 260 K. Solid lines are fitted to the data using the generalized HN equation and each dashed line stands for one individual contribution ($\sigma_{DC}$, $\alpha$ relaxation, $\beta$ relaxation and MWS process). The red symbols and lines correspond to $\varepsilon''$ while the blue ones relate to $\varepsilon'$.

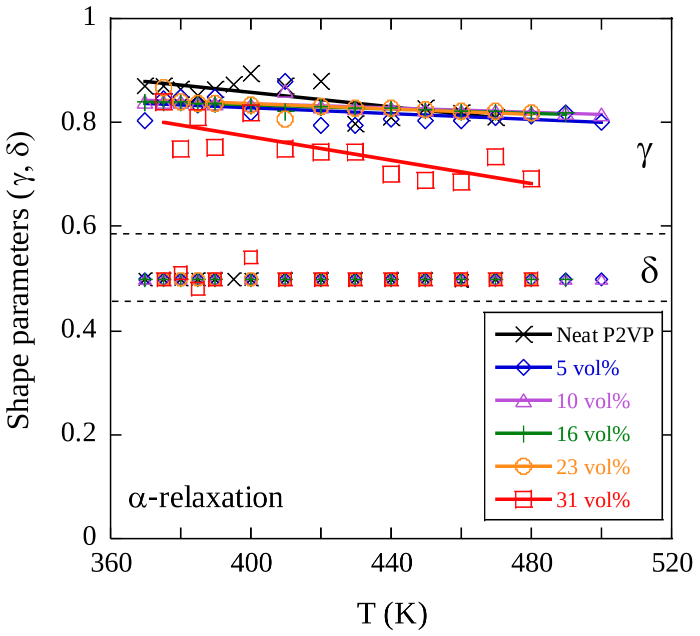


**Supplementary Figure 8.** HN function shape parameters of the $\alpha$-relaxation for the neat matrix and the composites loaded with 5, 10, 16, 23 and 31 vol% cast in a mixture of MEK and pyridine. Note that we fixed $\delta$ (asymmetric part) to be equal to 0.5 for almost all temperatures and all filler fractions considering the very narrow dispersion of $\delta$ in the free fits.

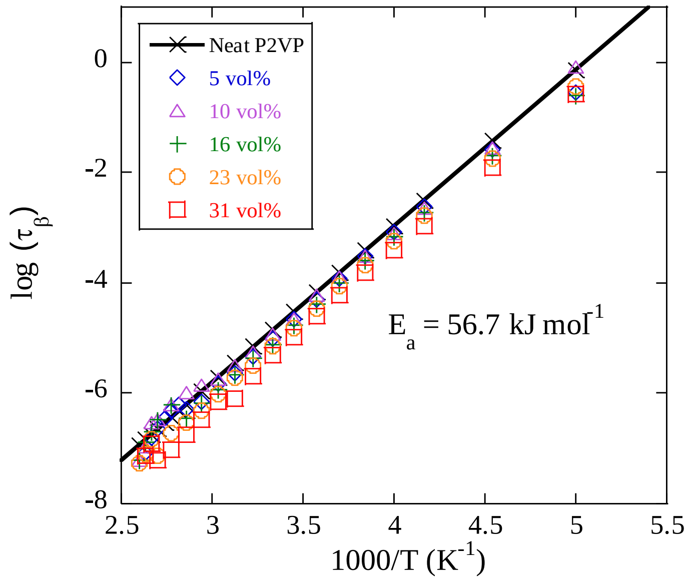


**Supplementary Figure 9.** $\beta$ relaxation time as a function of 1000/T for a neat P2VP ($M_w$ = 554 kg mol$^{-1}$) and composites loaded with 5, 10, 16, 23 and 31 vol% in silica cast in a mixture of MEK and pyridine.

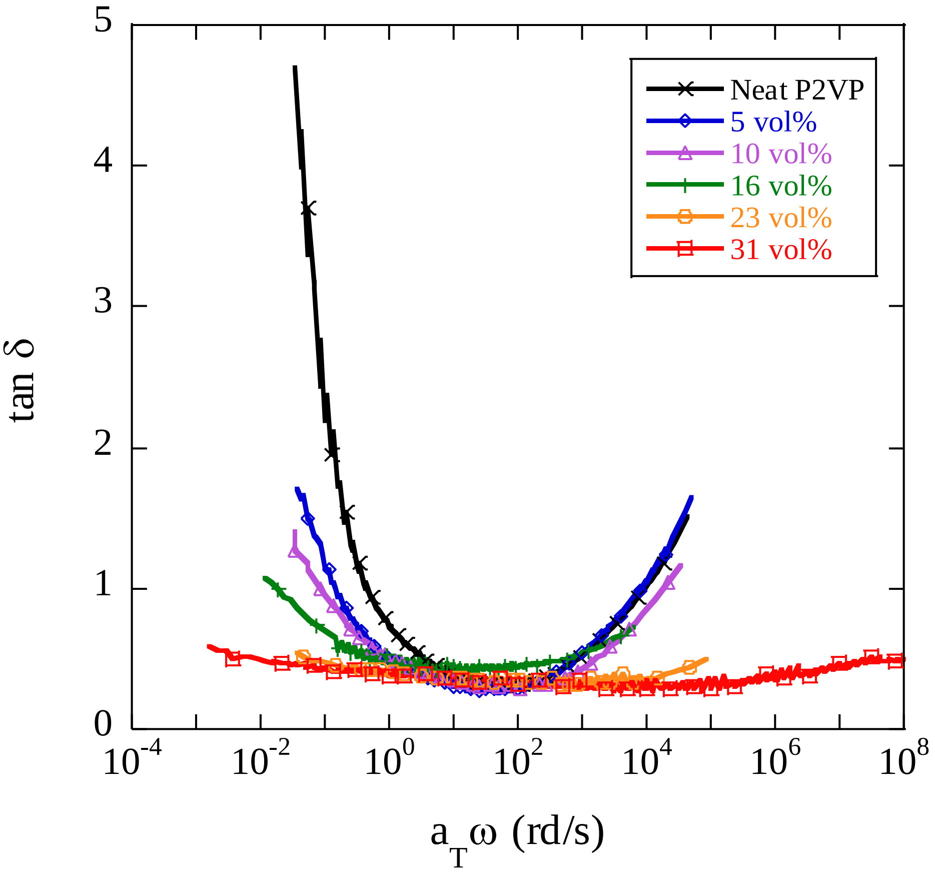


**Supplementary Figure 10.** tan($\delta$) master curves corresponding to the Fig. 3a in the article ($T_{ref}$ = 453 K), $M_w$ = 554 kg mol$^{-1}$.

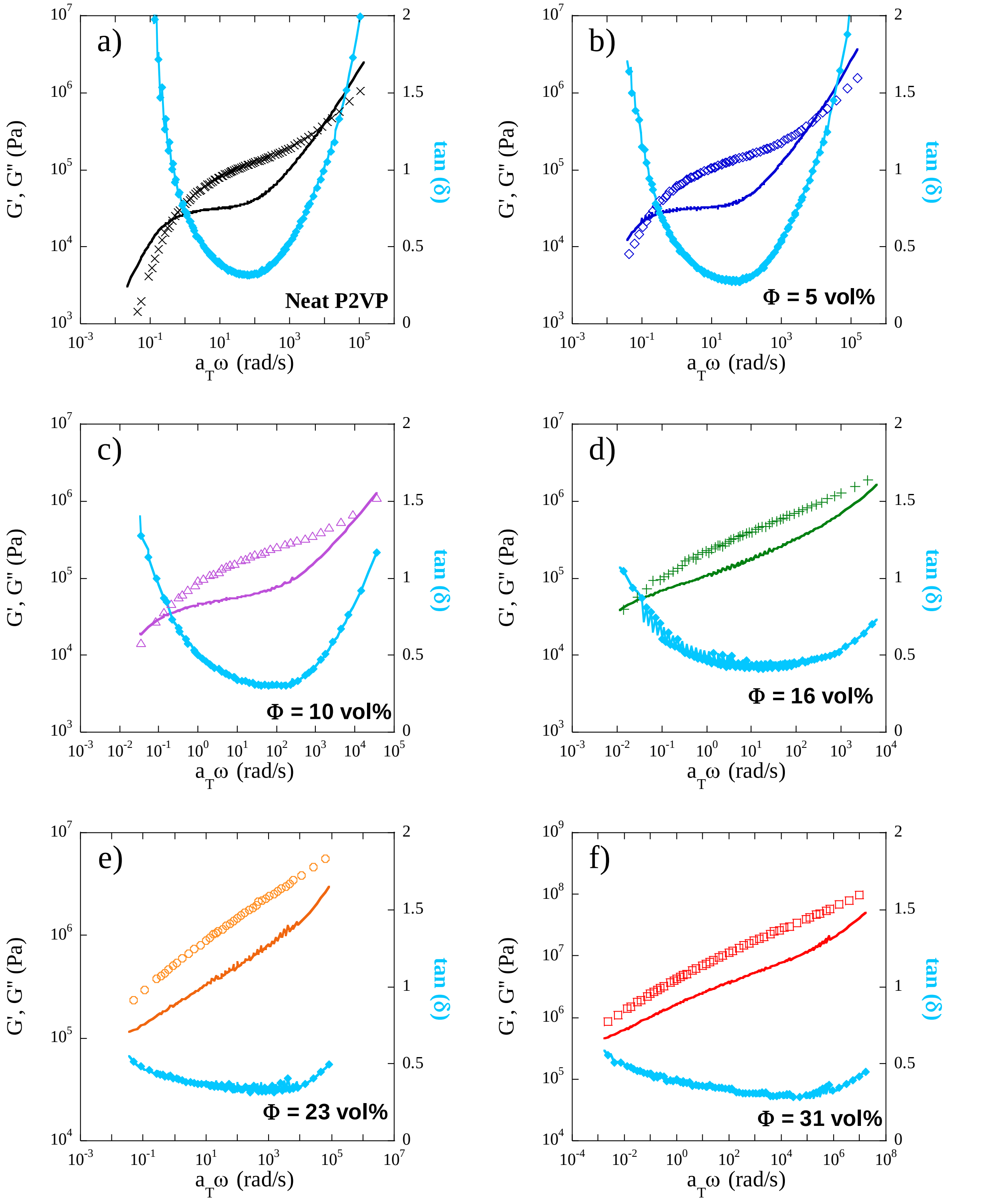


**Supplementary Figure 11.** ***G′***, ***G′′*** and tan($\delta$) master curves obtained from the tTS performed on ***G′*** and ***G′′*** for **a)** neat P2VP (554 kg mol$^{-1}$) and the nanocomposites loaded with **b)** 5 vol%, **c)** 10 vol%, **d)** 16 vol%, **e)** 23 vol% and **f)** 31 vol% silica NPs. The reference temperature is set to ***T*** = 453 K. As in the article, only horizontal shifting was used. The temperature range we utilized to build the mastercurve is 403 K-473 K, extended to 383 K-473 K for the 31 vol% sample.

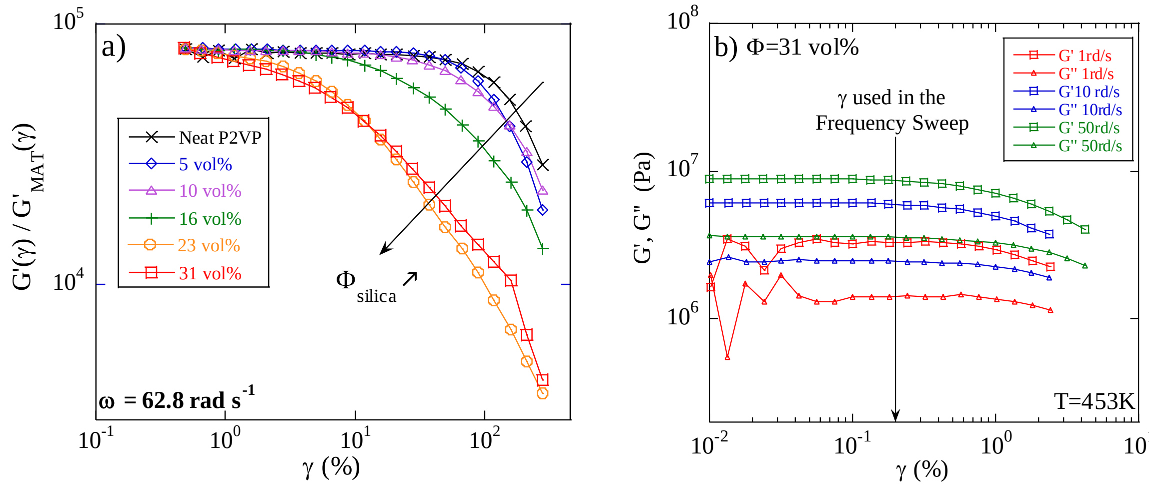


**Supplementary Figure 12. a)** Normalized dynamic strain sweeps on the series of composites ($M_w$ = 554 kg mol$^{-1}$) cast in a mixture of MEK and pyridine performed at 62.8 rad s$^{-1}$ at $T$ = 453 K. **b)** Strain sweeps performed on the 31 vol% sample at $\omega$ = 1, 10 and 50 rad s$^{-1}$ showing unambiguously the linear viscoelastic regime (performed with Anton Paar 702).

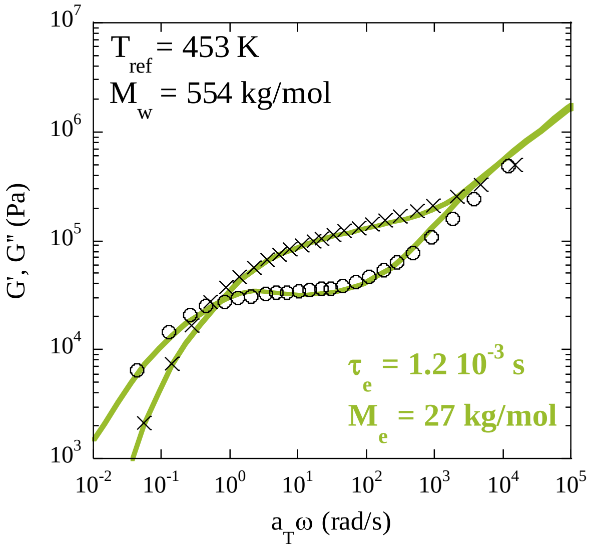


**Supplementary Figure 13.** $G'$ and $G''$ mastercurves for the neat P2VP with $M_w$ = 554 kg mol$^{-1}$ (as presented in the article). The solid lines are fits of the data using the linear theory developed by Likhtman and McLeish in 2002[10] with the software "Reptate". The model inputs are the temperature and the molecular weight allowing to extract the relaxation time of an entanglement strand ($\tau_e$ = 1.2×10$^{-3}$ s) as well as the molecular weight between entanglements. Note that in this case the latter has been forced to $M_e$ = 27 kg mol$^{-1}$ in order to match with the literature[11].

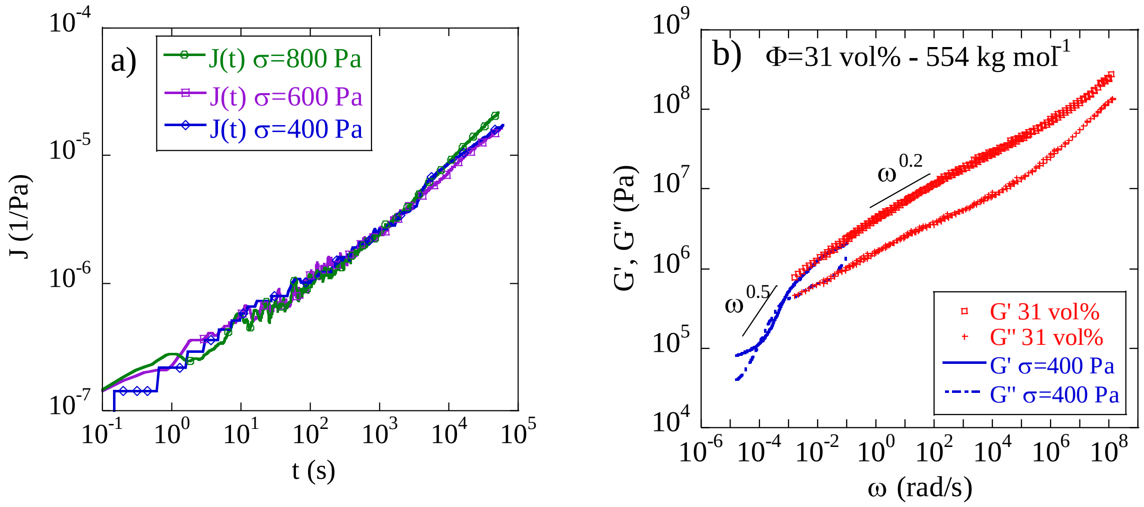


**Supplementary Figure 14. a)** Creep compliance as a function of the time for the sample loaded with 31 vol% measured with $\boldsymbol{\sigma}$ = 400 Pa (measured with DSR), $\boldsymbol{\sigma}$ = 600 Pa and $\boldsymbol{\sigma}$ = 800 Pa (measured with MCR 702) at $\boldsymbol{T}$ = 453 K. The data demonstrate that, unlike lower stresses, 800 Pa corresponds to nonlinear long-time response. **b)** $\boldsymbol{G'(\omega)}$, $\boldsymbol{G''(\omega)}$ for the nanocomposite loaded with 31 vol% in silica. The red symbols are identical to the data presented in Fig. 3a in the article while the lines are obtained by inverting the creep data presented in a) for $\boldsymbol{\sigma}$ = 400 Pa in the linear regime.

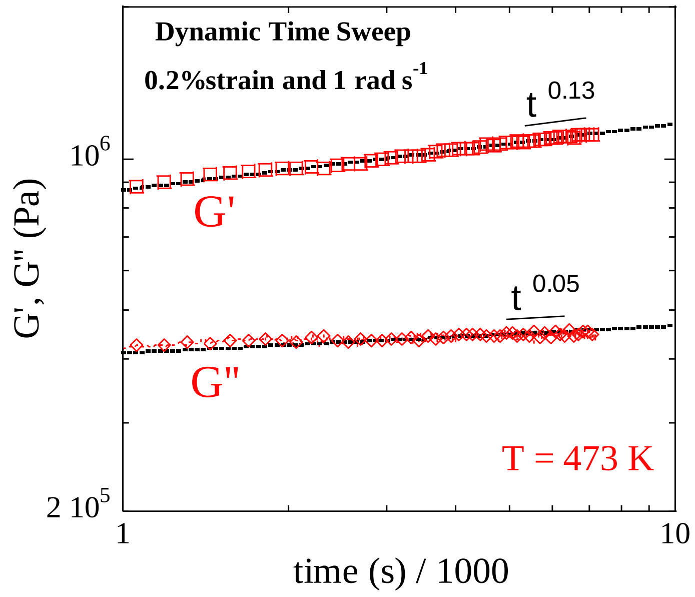


**Supplementary Figure 15.** Time sweep measurement performed at 473 K, 0.2% strain, 1 rad s$^{-1}$ on a sample made of P2VP (554 kg mol$^{-1}$) loaded with 31 vol% filler.

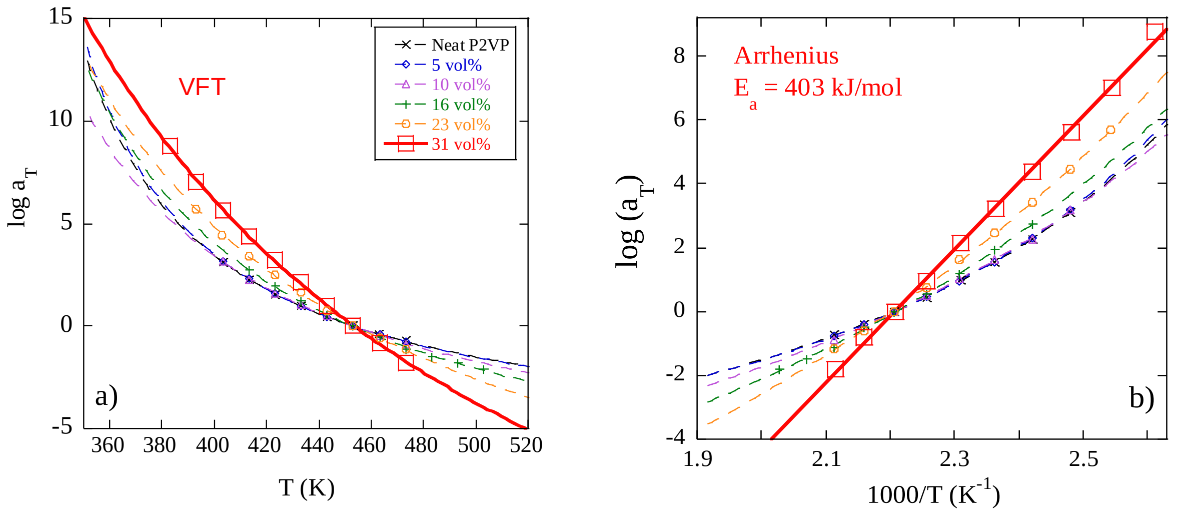


**Supplementary Figure 16.** Shift factor used to build the mastercurves for nanocomposites made of a 554 kg $mol^{-1}$ P2VP for different silica loadings as a function of **a)** *T* and **b)** 1000/*T*.

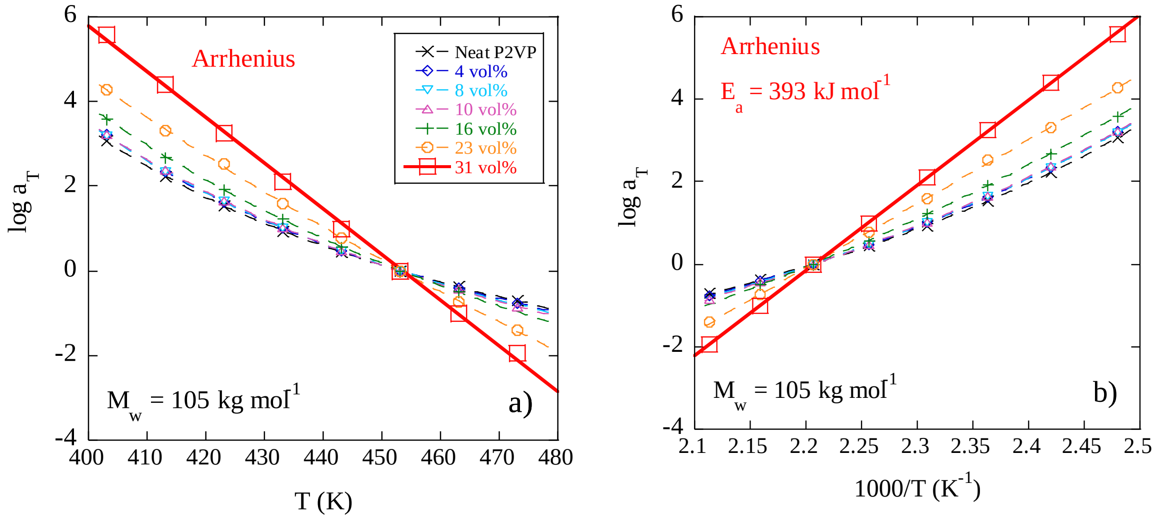


**Supplementary Figure 17.** Shift factor used to build the mastercurves for nanocomposites made of a 105 kg $mol^{-1}$ P2VP for different silica loadings as a function of **a)** T and **b)** 1000/T.

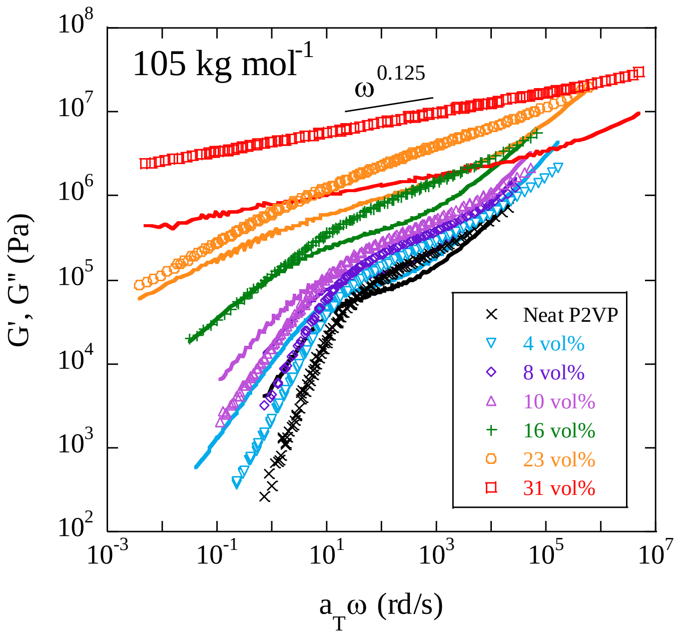


**Supplementary Figure 18.** Master curves of storage $G'$ and loss $G''$ moduli as a function of the frequency at $T_{ref}$ = 453 K for neat P2VP with $M_w$=105 kg mol⁻¹ and corresponding nanocomposites loaded with 4, 8, 10, 16, 23 and 31 vol%.

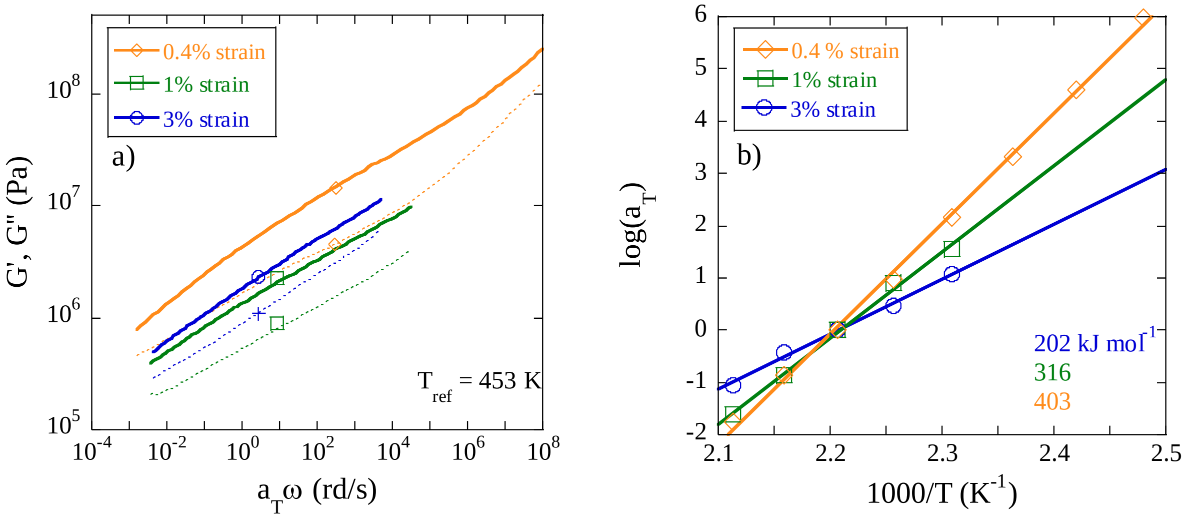


**Supplementary Figure 19. a)** Mastercurves for $G'$ and $G''$ for a nanocomposite loaded with 31 vol% in silica and $M_w$ = 554 kg mol$^{-1}$ for different strain amplitudes ($T_{ref}$ = 453 K). **b)** Corresponding shift factor and Arrhenius fit.

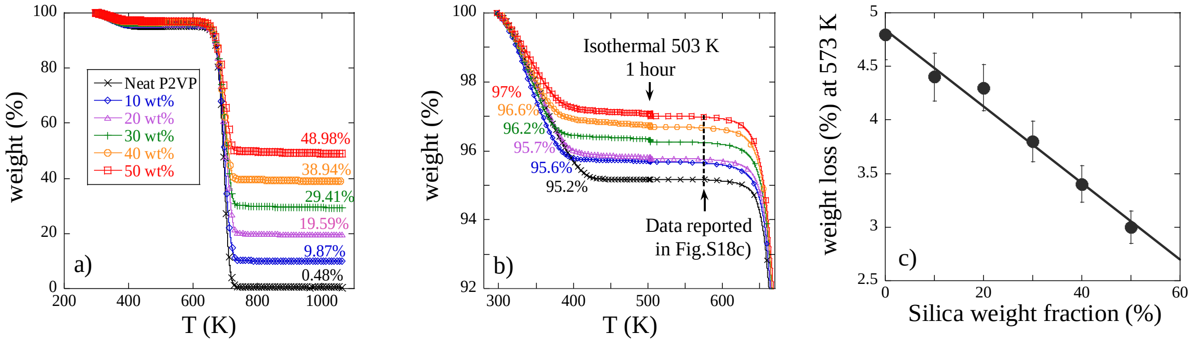


**Supplementary Figure 20. a)** TGA thermograms for the neat P2VP matrix and composites loaded with silica NPs (10, 20, 30, 40 and 50 weight% respectively equivalent to 5, 10, 16, 23 and 31 vol%) cast in a mixture of MEK and pyridine. **b)** Focus on the water evaporation. The loss in mass is higher when the silica fraction is lower suggesting that we are eliminating the water adsorbed within the P2VP (via H-bonds). Note that no significant loss in mass occurs during the isothermal treatment at 503 K (all the water is removed) **c)** Loss in mass measured at 573 K after staying isothermally at 503 K for one hour.

# Supplementary Tables

## Time Temperature Superposition

**Supplementary Table 1:** WLF and Vogel fit parameters

| vol% | $T_g$ | $T_\infty$ (Vogel, K) | $c_1$ (VFT) | $c_1$ (WLF) | $c_2$ (WLF, K) |
|---|---|---|---|---|---|
| 0 | 374.6 | 300.97 | 6.38 | 6.38 | 152.18 |
| 5 | 375.0 | 303.52 | 6.37 | 6.36 | 149.63 |
| 10 | 375.5 | 268.16 | 8.53 | 8.52 | 184.99 |
| 16 | 375.6 | 271.67 | 9.89 | 9.85 | 181.48 |
| 23 | 376.4 | 229.29 | 15.28 | 15.35 | 223.86 |
| 31 | 378.1 | 163.47 | 27.12 | 27.14 | 289.9 |

## Monte-Carlo Simulations

**Supplementary Table 2.** Conversion between weight fraction silica, volume fraction silica and packing fraction.

| Weight % NP | Vol% NP | $\eta_{NP}$ |
|---|---|---|
| 10 | 4.8 | 0.028 |
| 20 | 10 | 0.0595 |
| 30 | 16 | 0.095 |
| 40 | 23 | 0.136 |
| 50 | 31 | 0.183 |

**Supplementary Table 3.** Mean wall-to-wall distance between a silica NP and its $n^{th}$ closest neighbors ($n$=1, 2 and 3).

| Number density, $n$ | Packing fraction, $\eta$ | First neighbor (nm) | Second neighbor (nm) | Third Neighbor (nm) | Average three closest (nm) | Torquato (nm) | Geometrical estimate (nm) |
|---|---|---|---|---|---|---|---|
| 0.10 | 0.05 | 5.51 | 9.62 | 15.85 | 11.7 | 9.74 | 16.16 |
| 0.15 | 0.078 | 3.89 | 7.10 | 12.26 | 7.75 | 6.05 | 12.35 |
| 0.23 | 0.12 | 2.55 | 4.83 | 8.98 | 5.46 | 3.51 | 8.85 |
| 0.345 | 0.18 | 1.56 | 3.10 | 6.25 | 3.63 | 1.95 | 5.96 |
| 0.59 | 0.31 | 0.63 | 1.33 | 3.10 | 1.69 | 0.73 | 2.67 |

# Supplementary Notes

## Supplementary Note 1: Transmission Electron Microscopy (TEM)

Supplementary Fig. 1 presents micrographs of samples loaded with 5 vol% and 31 vol% silica NPs showing the good dispersion of the silica within the polymer phase for samples cast in a mixture of MEK and pyridine, even for the highly loaded films. Harton et al.[1] have complemented such TEM characterization with ultra-small angle X-ray scattering on the same system and shown that these random dispersion states occur across all scales that we can probe.

## Supplementary Note 2: Fourier Transform Infra-Red spectroscopy (FTIR)

FTIR was performed systematically on the full set of samples (bare silica, neat P2VP, 5, 10, 16, 23 and 31 vol%). We provide the full range spectra and peak assignments in Supplementary Fig.2.

While it is clear that hydrogen bonding increases with increasing filler fraction, (Supplementary Fig. 2a), one of the main issues in these experiments concerns the "multi-peak" observed close to 1000 $cm^{-1}$ which is known to be related with the different configurations of the Si-O bond. In fact, the literature reports that it may contain up to 9 contributions[2,3]. In Supplementary Fig.3, we show that it is possible to fit this data properly by using 4 Gaussian contributions, only changing their intensities and breadths. The contribution of the P2VP matrix is also added in the 5 and 10 vol% samples since it is not negligible.

The evolution of each contribution with the filler fraction (described in terms of number of monomer / number of silanol sites) is extracted and plotted in Supplementary Fig. 4. It shows that the "1100" contribution differs from the 3 others leading to a change in shape of the Si-O multi-peak in the global spectra shown in Supplementary Fig. 2c (the maximum position shifts from 1100 to 1040 $cm^{-1}$ with increasing silica content). Moreover, the strong intensity of the low-chemical shift contribution suggests that $Si\text{-}O^-$ groups might be created due to the proton delocalization caused by H-bonding with the pyridine nitrogen. The latter is clearly

observable through the broadening of the Si-O multi-peak when the silica content is increased (Supplementary Fig. 3f).

**Supplementary Note 3: 2D NMR**

While FTIR experiments provide evidence for H-bonding between the silica and the P2VP, it does not reveal any new chemical bonds. The 2D NMR presented in Supplementary Fig.5 confirms this important point showing that the pyridine ring signal in the nanocomposite loaded with 31 vol% remains unchanged with respect to the neat matrix (each proton in the ring corresponds to a particular carbon atom and no new peak is observed). Also, the ratio between different proton intensities remains unchanged.

**Supplementary Note 4: Differential Scanning Calorimetry (DSC)**

Differential scanning calorimetry measurements were performed using a TMDSC Q2000 from TA Instruments (USA) in the standard and modulated mode under continuous nitrogen flow. An identical thermal cycle was applied twice to all the samples in a standard mode consisting of (i) 5 minutes equilibration at 293 K; (ii) temperature ramp from 293 K to 453 K at 20 K $min^{-1}$; (iii) 5 minutes equilibration at 453 K and (iv) temperature ramp from 453 K to 293 K at 20 K $min^{-1}$. Subsequently, we switched to the modulated mode (±1 K every minute) to perform a temperature ramp from 393 K to 453 K at 2 K $min^{-1}$. The data shown in Fig. 1 in the article corresponds to this last step where the polymer specific heat capacity has been calculated following the protocol shown in the work of Holt et al.[4] using $c_p^{SiO_2} = 0.64 + 0.0015T$ J/K.

See the analogous measurement performed on series of nanocomposites based on a shorter P2VP ($M_w$ = 105 kg $mol^{-1}$, Supplementary Fig. 6). Note that the same trend is observed, i.e. a broadening of the glass transition (particularly at 31 vol%).

**Supplementary Note 5: Dielectric Relaxation Spectroscopy (DRS)**

For the dielectric spectroscopy measurements, a broadband high-resolution dielectric spectrometer (Novocontrol Alpha, Germany) was utilized in the frequency range from

$10^{-2}$ to $10^{7}$ Hz with temperature stability better than ±0.1 K. All the disk-shaped samples (30 mm in diameter, 0.15 mm in thickness) were annealed one hour at 473 K in the measurement cell to remove any possible water contribution (see TGA in Supplementary Fig. 20). Nitrogen was continuously blown in the cryostat to minimize polymer degradation and a small cross-shaped piece of PTFE was inserted with the samples in-between gold electrodes to avoid possible short-circuits due to polymer flow and maintain the electrodes spacing at 0.15 mm.

In addition to the well-known $\alpha$ (segmental) and $\beta$ (local) relaxations of polymers, dielectric spectroscopy reveals a Maxwell-Wagner-Sillars (MWS) process at low frequencies (high temperature) when silica is added. The latter is typical for nanocomposites as the interface between the polymer and the filler can generally be polarized. This contribution to the global dielectric response is particularly visible in the real part of the permittivity $\varepsilon'$ from $T$ = 400 K (see Supplementary Fig. 7a). The fitting procedure depicted there was thus realized with consideration of this process so that, in total, four contributions were described ($\alpha$, $\beta$, $\sigma$, MWS). Note that the electrode polarization “EP” at high temperature (low frequency) is excluded from the fitting procedure.

The Havriliak Negami (HN) function was used to fit simultaneously both real $\varepsilon'(\omega, T)$ and imaginary $\varepsilon''(\omega, T)$ parts of the complex dielectric permittivity in which we systematically tried to reduce the number of free parameters. As examples, the $\beta$ relaxation as well as the MWS process were fitted with the so called Cole-Cole HN term (*i.e.*, $\delta$ = 1) while we forced $\delta$ = 0.5 for the $\alpha$ relaxation description (see “asymmetric coefficient” $\delta$ in Supplementary Fig. 8). Also, when it was not possible to keep a parameter constant, we systematically tried to make it following a linear law with the temperature (see the “symmetric coefficient” $\gamma$ in Supplementary Fig. 8). In the HN function below $j$ stands for $\alpha$, $\beta$ or MWS:

$$\epsilon^*(\omega) = -i\frac{\sigma}{\varepsilon_0\omega} + \varepsilon_\infty + \sum_j \frac{(\Delta\varepsilon)_j}{\left(1 + \left(i\omega\tau_j\right)^{\gamma_j}\right)^{\delta_j}}$$

Prior to the fitting, a pre-treatment of the whole data set was applied in order to get rid of any artifacts coming from surface roughness or thickness variations. The latter

consisted of normalizing both $\varepsilon'(\omega, T)$ and $\varepsilon''(\omega, T)$ by applying a correction factor in order to have the high-frequency (low temperature) limit of the real part of the permittivity $\varepsilon_\infty$ equal to 2.53. Such a value is related to the refractive index ($n \approx \varepsilon_\infty{}^{0.5}$) of the P2VP ($n^{\mathrm{P2VP}} \approx 1.59$) which is found to be very close to silica ($n^{\mathrm{silica}} \approx 1.54$) justifying de facto a common normalization for different filler content. Additionally, this treatment allowed highlighting the broadening of the $\alpha$ relaxation with increasing the filler fraction through the decrease of the $\gamma$ parameter (see Supplementary Fig. 8) from ca. 0.9 (neat P2VP) to 0.7 (composites loaded with 23 vol% and 31 vol% silica NPs).

Similarly to the $\alpha$ relaxation characteristic time $\tau_\alpha$ (inset Fig. 2b in the article), $\tau_\beta$ did not show any significant evolution with silica content (Supplementary Fig. 9). The corresponding activation energy, close to 57 kJ mol$^{-1}$, falls well into the standard range for polymers, which is much smaller than that of the $\alpha$-relaxation ($\approx$ 400 kJ mol$^{-1}$) for highly loaded samples. One can remark however the slight decreasing of $\tau_\beta$ when the silica content increases. This very weak trend (acceleration) could be attributed to small changes in shape of the $\beta$-process ($\gamma$ parameter) and does not allow, alone, to conclude on the impact of silica NPs on the polymer local dynamics.

**Supplementary Note 6: Rheology**

Rheological measurements (Fig. 3 in the article and Supplementary Figs. 10-11-12a-13-14b-18-19) were performed in a strain controlled rheometer (ARES 2kFRTN1 from Rheometric Scientific, currently TA, USA) using stainless steel parallel plates of diameter 8 mm (about 0.5 mm in sample thickness) and a nitrogen convection oven (to maintain inert atmosphere) in the temperature range 473-403K with temperature control of ±1 K. Strain sweeps (Supplementary Fig. 12a) were performed at $T$ = 453 K and $\omega$ = 62.8 rad s$^{-1}$, $M_w$ = 554 kg mol$^{-1}$. Results presented in Supplementary Fig. 12b and Supplementary Fig. 14a have been obtained with a very sensitive stress-controlled Anton Paar 702 (Austria), that can provide accurate measurements also in strain controlled mode, hence allowing us to extend the measurements to low torques. Additional creep measurements were performed with another stress-

controlled rheometer (Rheometrics DSR, USA) to confirm the data (Supplementary Fig. 14a).

In order to better show the consistency of the time-temperature superposition presented in the article Fig.3a, we provide in Supplementary Fig. 10, the master curves for $\tan\delta = G''/G'$ as a function of the silica content. For even more clarity, we then replot this data together with the $G'$ and $G''$ mastercurves for each sample (Supplementary Fig. 11). These two figures show the flattening of $\tan\delta$ with increasing the silica content highlighting the transition from the linear polymer to a cross-linked network behaving like a gel.

We then address the question of the non-linearity. The unambiguous message from the dynamic strain sweep experiments depicted in Supplementary Fig. 12a is that, as the $SiO_2$ fraction increases, the linear regime is reduced. This phenomenon is well known in the literature as the "Payne effect". Supplementary Fig. 12b confirms that the dynamic frequency sweep tests (see Fig. 3 in the article) were performed in the linear viscoelastic regime even for the sample loaded with 31 vol% silica.

Besides, Supplementary Fig. 13 shows a fit of $G'$ and $G''$ for the neat P2VP ($M_w$ = 554 kg mol$^{-1}$) performed from the Likhtman and McLeish theory[10] showing that the P2VP we use has comparable properties (in particular the molar mass between entanglements $M_e$) as previously reported in the literature[11]. From $M_e$, we evaluate the tube diameter $a = bN_e{}^{1/2}$=15 nm.

In addition, in order to address the behavior at very low frequencies (not accessible with dynamic measurements due to temperature limitations to avoid degradation and prohibitively long measurement times at frequencies below $10^{-2}$ rad s$^{-1}$), we performed creep measurements with the 31 vol% sample. In Supplementary Fig. 14a, we present the evolution of the compliance as function of time, measured for values of shear stress $\sigma$ = 400 Pa, $\sigma$ = 600 Pa, and $\sigma$ = 800 Pa. In the last case, the higher compliance at long time suggests that the sample is slightly in the non-linear regime.

For $\sigma$ = 400 Pa and $\sigma$ = 600 Pa, however, despite the noisy data at short times, one can see that the tests are reproducible, suggesting linear response and enabling inversion (Supplementary Fig. 14b) in order to extend the frequency range of $G'$ and $G''$ to $10^{-5}$ $s^{-1}$. To do so, we used the software NLREG[5, 6] which, based on non-linear Tikhonov regularization methods, is suitable for solving nonlinear ill-posed inverse problems.

The creep measurements show that the linear viscoelastic moduli exhibit a cross-over of the power law exponent from 0.2 to 0.5. The weak power-law exponent of 0.2, which extends over a remarkable range of about 10 decades in frequency, is almost indistinguishable from a logarithmic dependence and may be considered as an effective plateau. However, the high plateau modulus of the composite with values in the range between 100 MPa and 1 MPa is clearly distinct from the rubbery plateau modulus of the P2VP matrix (Fig. 3 in the article). The very large composite modulus corresponds to very high density of stress-carrying objects, with size on the order of a monomer to several Kuhn segments. Hence, in this regime the dynamics reflects the motion of these short chain segments, which are slowed down by their strong interactions (adsorption) to the particles, and the modulus is $\sim kT/$(adsorbed chain segments). Increasing NP fraction enhances the value of the modulus as NPs are closer and the number of segments bridging them is reduced. The weak power law or nearly logarithmic shape of this regime can be explained by the fact that these segments are adsorbed along the surface as "trains" with different energies and an exponentially distributed spectrum of desorption times is needed in order for them to move. Hence, the weak slope is characteristic of this type of network with segment-particle bonds and should not depend significantly on molecular weight. This region reflects a broad distribution of desorption modes. Without such desorption, the whole composite would be practically "frozen". The data suggest that desorption rates are distributed over ~10 decades of frequency, implying a broad spectrum of different adsorption energies that differ from each other by $\sim kT \ln(10^{10}) \approx 23\ kT$. The fact that the relaxation spectrum is almost flat (0.2 slope) suggests that most segments are adsorbed/bound to the particles (either directly or through their chain neighbors) and are thus slowed down. The motion of larger chain sections requires simultaneous desorption of longer sub-sections (or stronger bound segments).

At lower frequencies (below $10^{-3}$ rad $s^{-1}$ in Supplementary Fig. 14b) the 0.5-slope indicates a different dynamics and it is tempting to associate it to sticky-Rouse[7]. Given the large molar mass of the P2VP, one may expect an entanglement plateau (lowered from pure melt) and sticky reptation at still lower frequencies[8], which is consistent with the available data. Though final assignment is premature based on these findings, it is safe to conclude that the observed dynamics is not due to percolation but to desorption of segments bound to NPs. Further work with more extended creep and creep recovery measurements is under study.
In order to complete the picture at long time, we performed a time sweep measurement at 473 K on the nanocomposite loaded with 31 vol% (Supplementary Fig. 15). The result reveals aging, a characteristic of glassy materials.

In Supplementary Table 1, one can remark that we provide VFT parameters for the 31 vol% data while it is fitted with an Arrhenius-like law in the article. This comes from the different representation we used and the global fit agreement we obtained. As an illustration, we present in Supplementary Fig. 16, the shift factor relative to the 31 vol% sample (554 kg $mol^{-1}$) as a function of $T$ and $1000/T$ respectively fitted with VFT and Arrhenius models. The important feature is however the strong evolution of the fit parameters (whatever the model) above 16 vol% in silica (see Supplementary Table 1) suggesting a transition in the dynamics.

In order to show that the Arrhenius picture is relevant, we also provide the same data extracted from nanocomposites made from a shorter P2VP (Supplementary Fig. 17, 105 kg $mol^{-1}$ instead of 554 kg $mol^{-1}$). Once again, the plot clearly shows that the dynamic of the nanocomposite loaded with 31 vol% is Arrhenius-like with a similar activation energy close to 400 kJ $mol^{-1}$. We believe that such a high value is related with the NPs bridging and has no direct connection with the glassy state of the P2VP (the apparent activation energy of the neat polymer at $T = T_g$ is found to be close to 500 kJ $mol^{-1}$ for both 105 and 554 kg $mol^{-1}$ samples).

The corresponding mastercurves for 105 kg $mol^{-1}$ based nanocomposites are provided in Supplementary Fig. 18. Here, we believe that the 0.125 slope, slightly different from the 0.2 slope observed for $M_w$ = 554, 219 and 36 kg $mol^{-1}$ is related to the sample preparation leading likely to a different filler structure.

Finally, we also show that the activation energy, extracted from 31 vol% samples, strongly depends on the strain applied to perform the series of frequency sweeps. Note that even at a strain amplitude of 1%, the 31 vol% sample behaves non-linearly (in agreement with Supplementary Fig. 12).

## Supplementary Note 7: Thermo-Gravimetric Analysis (TGA)

Thermogravimetric experiments presented here in Supplementary Fig. 20 were performed with a TGA-Q500 (TA instruments). All measurements were conducted under a high purity nitrogen flow on pre-dried samples according to the following protocol: (i) room temperature to 503 K at 5 K $min^{-1}$; (ii) isothermal at 503 K for one hour; (iii) 503 K to 1073 K at 20 K $min^{-1}$.

## Supplementary Note 8: Related to Table 1

Based on the shift factors $a_T$, we were able to study the system dynamics (see Fig. 4 in the article). In Supplementary Table 1, we provide the fit parameters extracted from WLF and VFT laws at $T_{ref}$ = 453 K. As expected, the Vogel temperature $T_\infty$ and $c_2$ (WLF) satisfy: $T_{ref} = T_\infty + c_2$.

# Supplementary Methods

## Monte-Carlo Simulations

Monte Carlo simulations were performed on hard sphere fluids with varying box size. Typically, we considered 1000 spheres each of unit diameter in a cubic box of side $L$. The number density $\rho^* = \frac{N}{L^3}$ is thus varied systematically in a series of simulations (see Supplementary Table 2). We simulated these samples by using random translations and then using the Metropolis algorithm- the major output is the mean value of distances to the first, second and third neighboring spheres as a function of density.

In the experimental analysis discussed to this point we calculated the vol% silica by using a density of 2.1 gm cm$^{-3}$ (amorphous silica) and 0.95 gm cm$^{-3}$ for the P2VP. The density of crystalline silica is ~ 2.65 gm cm$^{-3}$. If we reasonably assume that the crystal corresponded to a $\eta$ = 0.74 (FCC), where the packing fraction $\eta = \frac{\pi}{6}\rho^*$. This then yields that the $\eta$ for the amorphous silica is $\frac{0.74*2.1}{2.65} \approx 0.586$ which is consistent with the packing fraction known for glasses. We thus assume that the "hard core" density of the silica cores is $\frac{2.65}{0.74} = 3.58$ gm cm$^{-3}$. On this basis we can compute the packing fraction of silica at various weight% in the experimental nanocomposites.

In Supplementary Table 3 we report the mean nearest wall-to-wall distances between NPs and their $n^{\text{th}}$ neighbors as determined by the simulations. We note, in particular, that the distance between a particle and its closest neighbor becomes comparable with the P2VP Kuhn length for samples loaded with 31 vol%. This is believed to be the origin for the "glassy" features we observed for these highly filled samples (mainly Arrhenius dependence of the shift factor $a_{T},$ see Fig. 4 in the article).

# Supplementary References


1 Harton, S. E. *et al.* Immobilized Polymer Layers on Spherical Nanoparticles. *Macromolecules* **43**, 3415-3421 (2011).

2 Fidalgo, A. & Ilharco, L. M. The defect structure of sol-gel-derived silica/polytetrahydrofuran hybrid films by FTIR. *J. Non-Cryst. Solids* **283**, 144-154 (2001).

3 Innocenzi, P. Infrared spectroscopy of sol-gel derived silica-based films: a spectra-microstructure overview. *J. Non-Cryst. Solids* **316**, 309-319 (2003).

4 Holt, A. P. *et al.* Dynamics at the Polymer/Nanoparticle Interface in Poly(2-vinylpyridine)/Silica Nanocomposites. *Macromolecules* **47**, 1837-1843 (2014).

5 Weese, J. A Regularization Method for Nonlinear Ill-Posed Problems. *Comput. Phys. Commun.* 77, 429-440 (1993).

6 Honerkamp, J. & Weese, J. A Nonlinear Regularization Method for the Calculation of Relaxation Spectra. *Rheol. Acta* **32**, 65-73 (1993).

7 Chen, Q., Tudryn, G. J. & Colby, R. H. Ionomer dynamics and the sticky Rouse modela). *J. Rheol.* **57**, 1441-1462 (2013).

8 Leibler, L., Rubinstein, M. & Colby, R. H. Dynamics of reversible networks. *Macromolecules* **24**, 4701-4707 (1991).
